# Centralized Space Learning for open-set computer-aided diagnosis

**DOI:** 10.1038/s41598-023-28589-x

**Published:** 2023-01-30

**Authors:** Zhongzhi Yu, Yemin Shi

**Affiliations:** Beijing Academy of Artificial Intelligence Institution, Beijing, China

**Keywords:** Computational biology and bioinformatics, Diagnosis

## Abstract

In computer-aided diagnosis (CAD), diagnosing untrained diseases as known categories will cause serious medical accidents, which makes it crucial to distinguish the new class (open set) meanwhile preserving the known classes (closed set) performance so as to enhance the robustness. However, how to accurately define the decision boundary between known and unknown classes is still an open problem, as unknown classes are never seen during the training process, especially in medical area. Moreover, manipulating the latent distribution of known classes further influences the unknown’s and makes it even harder. In this paper, we propose the *Centralized Space Learning (CSL)* method to address the open-set recognition problem in CADs by learning a centralized space to separate the known and unknown classes with the assistance of proxy images generated by a generative adversarial network (GAN). With three steps, including known space initialization, unknown anchor generation and centralized space refinement, *CSL* learns the optimized space distribution with unknown samples cluster around the center while the known spread away from the center, achieving a significant identification between the known and the unknown. Extensive experiments on multiple datasets and tasks illustrate the proposed *CSL*’s practicability in CAD and the state-of-the-art open-set recognition performance.

## Introduction

**Figure 1:**
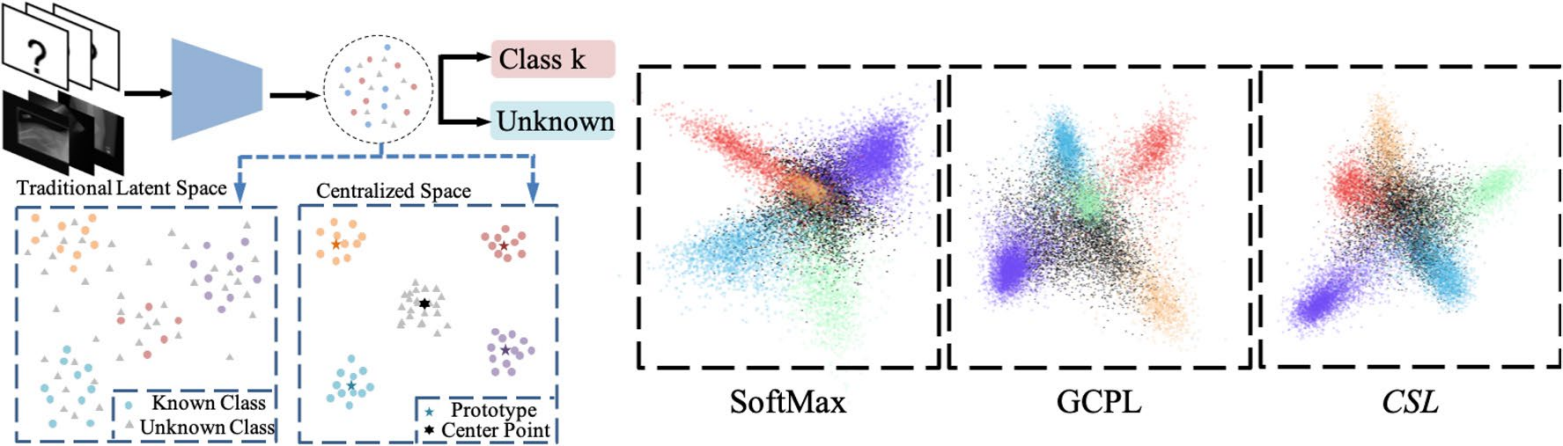
Left: open-set CAD task and *CSL*; right: the learned latent distribution. In the traditional latent space learnt by conventional methods, the network tends to embed all samples, including samples from unknown classes, around the cluster of known classes. This makes separating the known from unknown classes even harder. In *CSL*, we propose to learn a centralized space with a prototype for each known class and a center point in the center. All samples from known classes are clustered around the corresponding prototype and the samples from unknown classes are clustered around the center point.

With the rapid development of artificial intelligence, computer-aided diagnosis (CAD) has been widely used and achieves impressive performance^1–3^. However, it is a common assumption in existing CADs that the algorithm outputs a prediction from a set of predefined diseases, which is risky in some way. On the one hand, for difficult miscellaneous diseases or unknown diseases, brutally classifying them into one of the known diseases fails to make the correct diagnosis and may even delay the treatment leading to unacceptable results. On the other hand, given the massive workload in the radiology department, it is likely to have unqualified or part-mislabelled radiographs passing into the CAD system. Classifying the input radiograph under the closed-set assumption threatens the robustness of CADs. Hence, it is critical to equip CADs with open-set recognition ability to distinguish the unknown classes from the input samples, meanwhile preserving the classification ability on the known classes (for simplicity, in the remaining of this paper, we refer to samples from known and unknown classes as known samples and unknown samples, respectively). Left part of Fig. 1 presents a typical scenario of the open-set CAD problem^4^.

As one of the most widely used machine learning method, the convolutional neural networks (CNNs) based methods have achieved significant success^5–9^ and even beats the human-level performance in some specific tasks^10^. However, most works worked in a closed-set world and assumed all classes are known. As the conventional CNNs normally assume that all input samples belong to a set of predefined known classes, when exposed to samples from unknown classes, conventional CNN are forced to classify them into a known class. This defect severely deteriorates CNN’s performance in real world tasks and makes it un-robust and uncontrollable when applied in CAD scenarios.

As the unknown samples are never seen during the training process in open-set recognition, how to define the decision boundary between the known and unknown is the key problem. Existing open-set recognition methods tried to solve the task from the perspective of known classes and unknown classes. One of the most widely studied method is to learn the boundary of known class area so as to recognize the rest of the feature space as unknown. OpenMax^11^ proposed to change the Softmax layer into an extreme value theory (EVT) based layer. GCPL^12^ proposed the prototype learning approach, where the distance to the prototype is considered as the probability of a sample belonging to a known class. However, these approaches focus on manipulating the latent space of known samples and neglect the influence on the unknown classes. As shown in the right part of Fig. 1, the restriction on known latent distribution also makes the network tend to embed unknown samples around the known classes, deteriorating the network’s separation ability.

To alleviate the influence on unknown space, generative models are introduced into open-set recognition task to manipulate the unknown class boundary. In G-OpenMax^13^ and OSRCI^14^, the authors proposed to infer the unknown classes with generative models and forbid the network to recognize them as known. As it is impossible to precisely and efficiently manipulate the distribution of unknown classes, simply separating the generated fake images away from known classes cannot guarantee its effect on other unknown samples. Moreover, moving fake images away from one class may push them closer to another, which in turn worsens the separation. Given the goal of classifying the known samples and identifying the unknown samples, open set recognition algorithm needs to maximally separate the known samples from the unknown, meanwhile keeping the inter-class variance of known samples. Shu et al.^15^ proposed OpenGAN to augment the available set of real open training examples with adversarially synthesized “fake” data. However, such method still cannot make sure the generated samples belong to known classes.

Thus, on the one hand, the decision boundary between the known and unknown should be explicit and concise to alleviate the difficulty of latent distribution manipulation. On the other hand, the inter-class distance should be significant, while intra-class variance should be limited for known classes. The points on a hypersphere have the same distance to the center, while their inter-point distance can be easily controlled by radius. Therefore, we propose the *Centralized Space Learning (CSL)* framework to distinguish the unknown from known samples in a centralized space where the known classes are placed on a hypersphere and unknown classes clusters at the center of the sphere.

Our contributions are summarized as follows:

- Firstly, we introduce the concept of open-set CAD and discuss the key in separating the known and unknown samples. We argue the effectiveness of a concise and explicit decision boundary and propose a centralized space distribution to meet such requirements.
- Secondly, we propose a novel framework, *Centralized Space Learning*, for open-set CAD. In *CSL*, we propose to use three steps including known space initialization, unknown anchor generation and centralized space refinement to learn the centralized space that effectively distinguishes unknown samples from the knowns.
- Thirdly, extensive experiments show that the proposed *CSL* surpasses the state-of-the-art methods on open-set recognition tasks.

The remainder of this paper is organized as follows. “Related works” presents related works of *CSL*. “Methods” provides the detailed description of *CSL*. “Experiment” provides the implementation details, performance metrics, brief description of comparison methods and experimental results. “Conclusion” concludes the paper.

## Related works

The open set recognition task is first formally defined by Scheirer et al.^4^ in 2012, which aims to find out samples from unknown classes meanwhile preserving the model’s closed-set performance. Since then, many attempts have been made to solve it^11–14,16,17^. Existing approaches can be categorized into two types, discriminative model based methods and generative model based methods. The details are described in the following.

### Discriminative model based methods

In 2016, Bendale et al.^11^ proposed OpenMax to change the Softmax layer into an extreme value theory (EVT) based layer, opening the era of open set recognition with discriminative model. Shu et al.^18^ pushed the performance further by using *k*-sigmoid based technique. Hendrycks et al. proposed outlier exposure to introduce some of the unknown classes assisting in training. Yang et al.^12^ proposed GCPL, a prototype learning approach, where the distance to the prototype is considered as the probability of a sample belonging to a known class, and such method is widely used in open-set recognition. Attempts like OpenMax^11^ and CPL^12^ emphasized on modeling the latent distribution of known samples and separating samples that fall in abnormal locations, considering them as unknown classes. As metric learning aims to represent the similarity between samples with distance, some metric learning approaches can also be used in open set recognition problem, such as GCPL^12^ and HDML^19^. Zhang et al.^20^ proposed OpenHybrid framework, which encoded samples into a joint embedding space and detected unknown categories by a flow-based density estimator. Recently, Vaze et al.^21^ demonstrated that the ability of a classifier to find the unknown samples is highly correlated with its accuracy on the closed-set classes. This discovery also proves the importance of discriminative model based methods.

### Generative model based methods

Generative model based approaches proposes to deal with the open-set recognition problem with the assistance from generative models. Ge et al.^13^ proposed G-Openmax to introduce the concept of generative model based method into the open-set field. Then OSRCI method^14^ moved further by introducing the concept of counterfactual images. Inspired by the success of sparse reconstruction, a series of reconstruction based approaches have been proposed recently, such as C2AE^22^ and CROSR^16^. Shu et al.^15^ proposed OpenGAN to augment the available set of real open training examples with adversarially synthesized “fake” data. Girish et al.^23^ proposed that all GANs leave fingerprints on their generated images and it is possible to discover images generated from previously unseen GANs. Instead of using GANs, Zhou et al.^24^ proposed PROSER to learn placeholders for unknown classes in the embedding space.

## Methods

**Figure 2:**
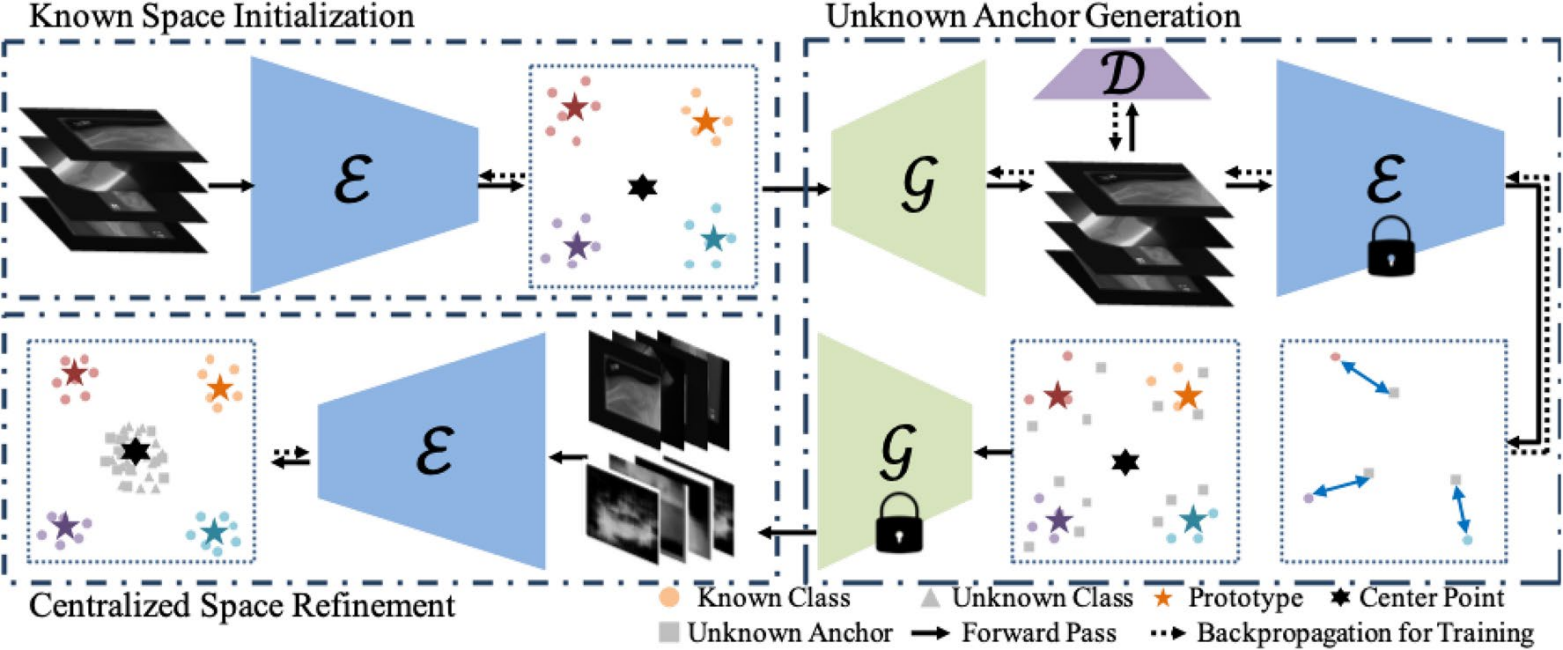
Framework of centralized space learning. In *Known Space Initialization*, $\mathscr {E}$ is trained to embed all known samples away from the center. In *Unknown Anchor Generation*, $\mathscr {G}$ and $\mathscr {D}$ are trained to generate proxy images. Then, $\mathscr {G}$ is fixed and used to generate proxy images. In *Centralized Space Refinement*, $\mathscr {E}$ is finetuned with proxy images and known samples to refine the centralized space.

In *CSL*, we aim to learn a centralized latent space with a center point in the center and prototypes of each known class around the center point. Samples from known classes are clustered to their corresponding prototypes, while unknown samples are clustered near the center point. The sample’s distance to the closest prototype is the criterion. Specifically, if the distance is below a threshold, the sample is considered belonging to the corresponding class, otherwise it is considered as unknown. Our *CSL* consists of three stages: known space initialization, unknown anchor generation and centralized space refinement. The overall framework of *CSL* is shown in Fig. 2.

In the remaining of this paper, we use the following notations. Given *N*-known-sample set $\{\varvec{X}_i\}_{i=1}^N$ in *K* known classes $\{1,2,\cdots ,K\}$ and a *d* dimensional latent space $\mathscr {O}\in \mathbb {R}^d$. For simplicity, we use $\varvec{X}_i \in k$ to represent $\varvec{X}_i$ belongs to class *k*. We define a set of *K* learnable prototypes $M = \{\varvec{m}_i\in \mathbb {R}^d, \ i\in \{1,2,\cdots , K\}\}$ in the latent space with each of them corresponding to a known class. Then we define the center point $\mu$ of latent space $\mathscr {O}$ according to the value of *M* as $\mu = \frac{1}{K}\sum _{i=1}^K\varvec{m}_i$ and $\mu$ is updated every time *M* updated.

### Known space initialization

In known space initialization, the discriminative embedding network learns a mapping to the latent space to cluster the known samples around the prototypes meanwhile spread prototypes away from $\mu$. After the centralized space is initialized, the vacuum around $\mu$ is prepared for the unknown samples.

With parameters in the discriminative embedding network $\mathscr {E}$ (for simplicity, we use $\mathscr {E}(\cdot )$ to represent the forward pass of $\mathscr {E}$) and *M* as trainable parameters, our objective in the known space initialization is

1 $$\begin{aligned} \begin{aligned} {} &\min _{\mathscr {E},M}\sum _{k=1}^{K}\sum _{\varvec{X}_i\in k}\kappa (m_k, \varvec{X}_i)- \rho _1\sum _{k=1}^K\kappa (m_k, \mu ) \\&\quad - \rho _2\sum _{k=1}^K\sum _{l=1}^K\kappa (\varvec{m}_k, \varvec{m}_l), \end{aligned} \end{aligned}$$

where $\kappa (,)$ is a measurement of distance, $\rho _1$ and $\rho _2$ are used to balance these factors.

Three losses including distance based cross entropy (DCE) loss, prototype loss and separation loss are proposed to initialize the space.

DCE loss aims to push samples away from unmatched prototypes and close to the correct one to increase the inter-class distance meanwhile slightly reduce the intra-class variance. Given $\varvec{X}_i\in k$, DCE loss is defined as

2 $$\begin{aligned} \begin{aligned} \mathscr {L}_{DCE}(\varvec{X}_i, k; \mathscr {E}, M)&= -\text {log}\ p(k\vert \varvec{X}_i) \\&= - \text {log}\frac{e^{-\gamma d(\mathscr {E}(\varvec{X}_i), \varvec{m}_k)}}{\sum _{j=1}^K e^{-\gamma d(\mathscr {E}(\varvec{X}_i), \varvec{m}_j)}}, \end{aligned} \end{aligned}$$

where $d(\mathscr {E}(\varvec{X}_i), \varvec{m}_k) = \Vert \mathscr {E}(\varvec{X}_i) - \varvec{m}_k\Vert _2^2$ is the square of $\ell _2$ distance between the sample $\varvec{X}_i$ and corresponding prototype $\varvec{m}_k$ in the latent space, $\gamma$ is the hyper parameter that controls the hardness of assignment.

Prototype loss aims to efficiently reduce the intra-class variance, clustering samples closer to their corresponding prototype, which is defined as

3 $$\begin{aligned} \mathscr {L}_P(\varvec{X}_i, k; \mathscr {E}, M) = \Vert \mathscr {E}(\varvec{X}_i) - \varvec{m}_k\Vert _2^2. \end{aligned}$$

As shown in Fig. 1, when the known classes are embedded into the latent space without any restriction, they are likely to be located randomly. As a consequence, by pushing the unknown away from a known class, the unknown samples are possibly moving close to another known class. An ideal way to avoid this situation is to embed all known classes in a pre-defined space, e.g., around a hypersphere centered at $\mu$. Therefore, the separation loss tries to restrict the prototypes on hypersphere, enlarge the radius of hypersphere and keep space near $\mu$ vacant. It is defined as

4 $$\begin{aligned} \mathscr {L}_{sep}(\mathscr {E}, M) = \max _{\forall i \in \{1,2,\cdot ,K\}} -\text {log}\Vert \varvec{m}_i - \mu \Vert _2^2 . \end{aligned}$$

This helps the *CSL* learn an embedding that all known samples are located around $\mu$, with a vacant area near *c*. When a prototype is located close to $\mu$, $\mathscr {L}_{sep}$ keeps pushing it away. Until all prototypes are of almost the same distance to $\mu$, $\mathscr {L}_{sep}$ pushes them successively.

The overall loss function in the known space initialization is

5 $$\begin{aligned} \begin{aligned} \mathscr {L}_{KSI}&= \mathscr {L}_{DCE}(\varvec{X}_i, k; \mathscr {E}, M) + \alpha _1 \mathscr {L}_{P}(\varvec{X}_i, k; \mathscr {E}, M) \\&\quad + \beta _1\mathscr {L}_{sep}(\mathscr {E}, M), \end{aligned} \end{aligned}$$

where $\alpha _1$ and $\beta _1$ are hyper parameters balancing the losses.

### Unknown anchor generation

*CSL* generates proxy images that are also embedded near *M* but having very small probabilities of belonging to any known classes to get a glimpse of the misclassified unknown samples. We call such generated proxy images in the latent space as unknown anchors. We propose to use a GAN to generate proxy images and unknown anchors. Given $\mathscr {E}$ and $\{\varvec{X}_i\}_{i=1}^N$ with distribution $\varvec{X} \sim p_{data}(\varvec{X})$, *CSL* trains the generator $\mathscr {G}$ and discriminator $\mathscr {D}$ to learn a mapping from the latent space $\mathscr {O}$ to the image domain $\mathscr {X}$, with input $\mathscr {E}(\varvec{X})$ to $\mathscr {G}$, $\mathscr {E}(\mathscr {G}(\mathscr {E}(\varvec{X}_i)))$ should be close to $\mathscr {E}(\varvec{X}_i)$. ($\mathscr {G}(\cdot )$ and $\mathscr {D}(\cdot )$ represent the forward pass.)

Thus, we train GAN with adversarial losses in^25^ and an additional cycle consistency loss similar to^26^, which is defined as

6 $$\begin{aligned} \mathscr {L}_{cyc}(\varvec{X}_i, \mathscr {E}; \mathscr {G}) = \Vert \mathscr {E}(\mathscr {G}(\mathscr {E}(\varvec{X}_i))) - \mathscr {E}(\varvec{X}_i) \Vert _2. \end{aligned}$$

Hence, the overall loss functions for $\mathscr {G}$ and $\mathscr {D}$ are

7 $$\begin{aligned} \begin{aligned} \mathscr {L}_{\mathscr {G}}(\varvec{X}, \mathscr {E}, \mathscr {D}; \mathscr {E})&= \mathscr {L}_{gen}(\varvec{X}, \mathscr {E}, \mathscr {D}; \mathscr {G}) + \lambda \mathscr {L}_{cyc}(\varvec{X}_i, \mathscr {E}; \mathscr {G}) \\ \mathscr {L}_{\mathscr {D}}(\varvec{X}, \mathscr {E}, \mathscr {G}; \mathscr {D})&= \mathscr {L}_{dis}(\varvec{X}, \mathscr {E}, \mathscr {G}; \mathscr {D}), \end{aligned} \end{aligned}$$

where $\mathscr {L}_{gen}(\varvec{X}, \mathscr {E}, \mathscr {D}; \mathscr {G})$ and $\mathscr {L}_{dis}(\varvec{X}, \mathscr {E}, \mathscr {G}; \mathscr {D})$ are adversarial loss defined in^25^, $\lambda$ is a hyper parameter balancing two losses. Parameters in $\mathscr {E}$ and *M* are fixed during training.

Then, we generate unknown anchors $\{\hat{\varvec{f}}_i\}_{i=1}^N$ and corresponding proxy images $\{\mathscr {G}(\hat{\varvec{f}}_i)\}_{i=1}^N$ with fixed $\mathscr {G}$ and $\mathscr {E}$. For each initial anchor $\varvec{f}_{\text {init}}$, they have a probability $P_f$ that $\varvec{f}_{\text {init}}\in \{\hat{\varvec{f}}_i\}_{i=1}^N$. $\varvec{f}_{\text {init}}$ and $P_f$ are defined as

8 $$\begin{aligned} \begin{aligned} P_f&= \min \left( \min \left\{ \frac{1}{r}\Vert \varvec{f}_{init} - \varvec{m}_k\Vert _2\right\} _{k=1}^K, 1\right) , \\&\qquad \text {where }\varvec{f}_{\text {init}} = \mathscr {E}(\varvec{X}_i) + \delta \end{aligned} \end{aligned}$$

where $\delta \in \mathbb {R}^d$ is a random vector with $\Vert \delta \Vert _2\in (0, 0.2]$, *r* is a threshold which is set to be larger than $70\%$ of samples’ $\ell _2$ distance to the nearest $\varvec{m}_i\in M$.

As the closer $\varvec{f}_{init}$ to *M*, the higher chance it belongs to the known class, we select $\varvec{f}_{init}\in \hat{\varvec{f}}$ with less probability. Thus, the majority of $\hat{\varvec{f}}$ are images that do not belong to any known classes. We keep generating until the number of unknown anchors equals to the training images.

### Centralized space refinement

During centralized space refinement, $\mathscr {E}$ is further refined in the centralized space, clustering the unknown anchors near $\mu$ and separating unknown from known samples. The objective function is

9 $$\begin{aligned} \begin{aligned} \min _{\mathscr {E}, M}&\sum _{k=1}^K\sum _{\varvec{X}_i\in k}\kappa (m_k, \varvec{X}_i) - \xi _1 \sum _{k=1}^K \kappa (m_k, \mu ) \\&\quad - \xi _2 \sum _{k=1}^K\sum _{l=1}^K\kappa (\varvec{m}_k, \varvec{m}_l) + \xi _3 \sum _{i=1}^N\kappa (\hat{\varvec{f}}_i, \mu ), \end{aligned} \end{aligned}$$

where $\xi _1$, $\xi _2$ and $\xi _3$ are used to balance these factors. We use the generated proxy sample and anchor pairs $(\mathscr {G}(\hat{\varvec{f}}), \mathscr {E}(\mathscr {G}(\hat{\varvec{f}})))$ to assist with the refinement.

We propose the cluster penalty to cluster $\{\hat{\varvec{f}}_i\}_{i=1}^N$ around $\mu$, which is defined as

10 $$\begin{aligned} \begin{aligned} \mathscr {L}_c&(\hat{\varvec{f}}, \mathscr {G},\mu , M; \mathscr {E}) = -\text {log}\frac{e^{-\gamma d(\mathscr {E}(\mathscr {G}(\hat{\varvec{f}})), \mu )}}{e^{-\gamma (d(\mathscr {E}(\mathscr {G}(\hat{\varvec{f}})), \mu ) + \sum _{i=1}^K d(\mathscr {E}(\mathscr {G}(\hat{\varvec{f}})), \varvec{m}_i))}}. \end{aligned} \end{aligned}$$

DCE loss, prototype loss and separation loss are also used during the refinement. The overall loss function is

11 $$\begin{aligned} \begin{aligned} \mathscr {L}_{CSR}&= \mathscr {L}_{DCE}(\varvec{X}, k; \mathscr {E}, M) + \alpha _2 \mathscr {L}_P(\varvec{X}, k; \mathscr {E}, M) \\&\quad + \beta _2 \mathscr {L}_{sep}(\mathscr {E}, M) + \theta \mathscr {L}_c(\hat{\varvec{f}}, \mathscr {G},\mu , M; \mathscr {E}), \end{aligned} \end{aligned}$$

where $\alpha _2$, $\beta _2$ and $\theta$ are hyper parameters balancing the losses.

## Experiment

### Experiment setup

#### Datasets and metric

**Figure 3:**
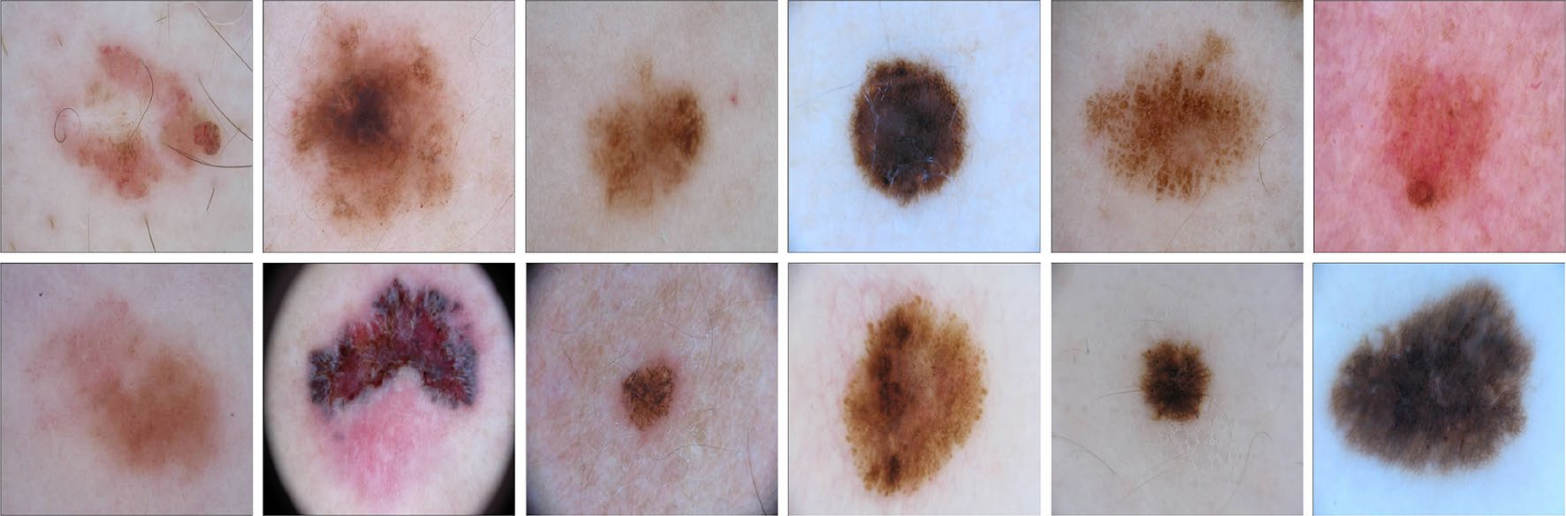
Sample images from ISIC-open dataset.

We evaluate the performance of the proposed *CSL* on two significant and common tasks, body part open-set recognition and open-set skin disease identification.

For body part open-set recognition, we use the radiograph dataset MURA^27^ by Stanford University, which has 40,561 images. Each of seven body parts is set as the unknown class with the remaining as known class iteratively.

For open-set skin disease identification, we propose a new dataset ISIC-open consisting of skin disease images collected from the International Skin Imaging Collaboration (ISIC) project^28^. Some of the sample images from the ISIC-open dataset are exhibited in Fig. 3. The ISIC-open dataset consists of 7491 images with a size of $256\times 256$ from 17 kinds of skin lesion with an imbalanced distribution. The proposed dataset is fine-grained given the similarity between different skin diseases, which increases the difficulty in recognition. The size of categories range from 1 to 1000. We select 6 categories with the most samples as known classes (5489 samples for training and 1410 samples for validation) and consider the others as unknown (592 samples).

We use F-measure^29^ and AUROC^30^ as the performance metrics. To compute F-measure and AUROC, we interpret a sample as positive when it is considered belonging to unknown class and interpret all other samples as negative. Then, F-measure and AUROC scores are computed similar to binary classification task.

#### Implementation details

We use ResNet18^8^ as the backbone of the discriminative embedding network $\mathscr {E}$. For $\mathscr {G}$ and $\mathscr {D}$, we use two nine-layer CNNs. In known space initialization, $\alpha _2 = 0.1$ and $\beta _1 = 0.1$. $\mathscr {E}$ is trained for 50 epochs with $lr=0.0001$. In unknown anchor generation, $\lambda =1$, $\delta = 0.1$. Both $\mathscr {G}$ and $\mathscr {D}$ are trained with $lr=0.0001$ for $2\times 10^4$ iterations. In centralized space refinement, $\alpha _2 = 0.1$, $\beta _2 = 0.01$ and $\theta = 0.1$. $\mathscr {E}$ is trained for 20 epochs with $lr=0.00005$. Adam optimizer^31^ is used in all experiments.

### Experiment results

#### Body part open-set recognition

**Figure 4:**
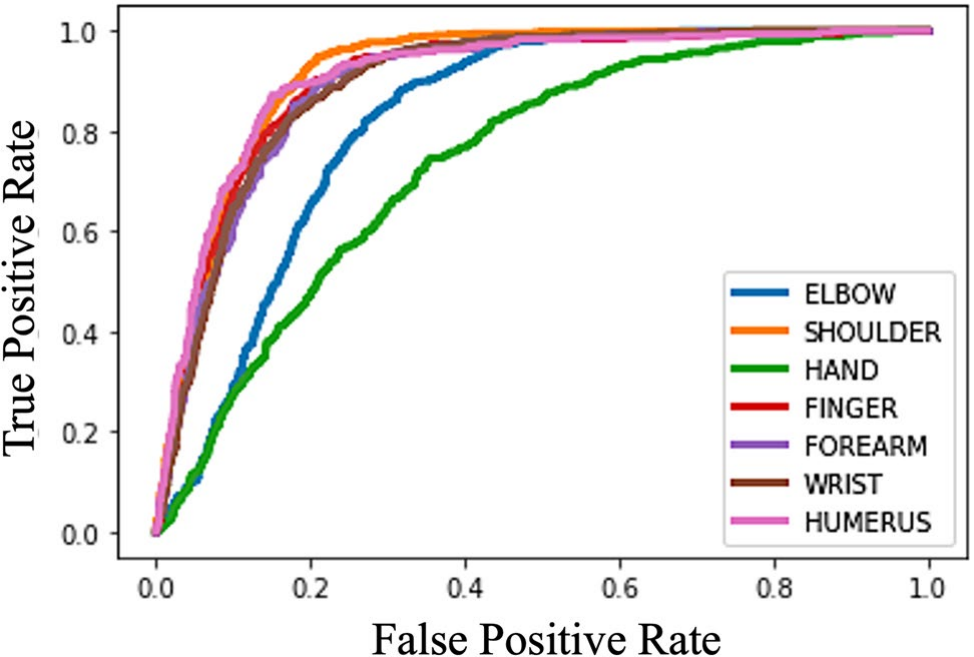
The ROC curves for all body parts on MURA dataset.

**Table 1:** The AUROC and F1 scores of body part open-set recognition on MURA dataset.

Method	SoftMax	OSRCI^14^	GCPL^12^	HDML^19^	RPL^32^	ARPL^33^	*CSL*
AUROC	0.734	0.755	0.853	0.855	0.873	0.871	0.872
F-Measure	0.761	0.770	0.841	0.841	0.86	0.861	0.851

For body part open-set recognition, benchmark and the state-of-the-art algorithms including OSRCI^14^, GCPL^12^, HDML^19^, RPL^32^ and ARPL^33^ are used for comparison. The results are shown in Table 1. The “SoftMax” method means training a softmax classifier with the same backbone network and recognizes a sample as unknown when all confidence scores are below threshold. The proposed *CSL* has suppressed most comparison algorithms with a solid margin, proving the effectiveness of *CSL* in separating the unknown from the known. Comparing with RPL and ARPL methods, CSL has a similar AUROC while having a lower F-Measure. We believe that such result is related to the choice of threshold. The assumption is proved on the ISIC-open dataset where CSL achieves much better AUROC and significant better F-Measure. Comparing to OSRCI which also uses the generative model, we ascribe the performance improvement to the centralized space to separate the unknown and known. This also proves our argument that the explicit and concise boundary enhances the open-set recognition ability. The improvement over the GCPL further proves the effectiveness of the centralized space. Since GCPL is also a prototype learning based method, but without the concept of centralized space and space refinement, the superiority in performance of *CSL* proves the effectiveness of introducing the unknown anchor and proxy image during refinement. The ROC curves for each body part are shown in Fig. 4. It is clear that some parts have very different distribution. For example, the open-set performance of “HAND” is far lower than all other parts. We claim it to be the results of hands being shared by several other parts (e.g. finger, wrist), which makes it hard to distinguish hand from other parts.

#### Open-set skin disease identification

We further evaluate the proposed *CSL*’s ability on open-set skin disease identification using the ISIC-open dataset. Following the protocol in CADs, we use the pretrained ResNet18 as the backbone for all algorithms in the experiments. For comparison, multiple benchmark and state-of-the-art algorithms in open-set recognition and metric learning are used, including G-Openmax^13^, OSRCI^14^, HDML^19^, RPL^32^ and ARPL^33^. The results are shown in Table 2. The proposed *CSL* has suppressed all the comparison algorithms in both AUROC and F-measure, proving the efficiency in separating the unknown diseases of the proposed *CSL* algorithm. Comparing to HDML, which also generates the anchor in latent space but do not introduce the concept of explicit separation, the performance improvement suggests that the latent distribution is critical in separation. Comparing to the current state-of-the-art methods, RPL and ARPL, CSL achieves significant better AUROC and F-Measue scores.

**Table 2:** The results of open-set skin disease identification on ISIC-open dataset.

Method	SoftMax	G-Openmax^13^	OSRCI^14^	HDML^19^	RPL^32^	ARPL^33^	*CSL*
AUROC	0.688	0.703	0.713	0.730	0.631	0.672	0.752
F-Measure	0.681	0.699	0.709	0.726	0.425	0.586	0.736

### Ablation study

To test the contribution of each module, we conduct experiments on one split in the MURA^27^ dataset and use AUROC^30^ as the metric to evaluate the performance.

**Table 3:** Latent space initialization comparison.

Method	AUROC
SoftMax	0.753
K+1	0.805
RO	0.821
$\textit{CSL}_{init}$	0.859
*CSL*	**0.894**

Significant values are in [bold].

#### Different initialization

We first evaluate the necessity of known space initialization. For comparison, we train the network under the following settings and the results are shown in Table 3. In K + 1, the model is first trained with known samples using softmax and then use the generated proxy images as an additional class to refine the model. In Refine-Only (RO), we only perform centralized space refinement with procedures exactly the same as in “Centralized space refinement” without known space initialization and the unknown anchors are randomly generated to generate proxy images. For $\textit{CSL}_{init}$, we only perform the known space initialization without unknown anchor generation and centralized space refinement. From Table 3, it is obvious that lacking the proper known space initialization has severe damage to the network’s performance. Both Refine-Only and $K+1$ are much worse than *CSL*. Also, the impressive performance of $\textit{CSL}_{init}$ demonstrates the superiority of the latent distribution adopted in *CSL*.

**Table 4:** Latent space distribution comparison.

Distribution	AUROC
Random	0.849
Uniform	0.853
C-Cent.	0.874
*CSL*	**0.894**

Significant values are in [bold].

#### Latent space distribution

We also argue that, by designing explicit and concise distribution, the unknown samples’ latent distribution can be manipulated easier. Hence, we tested other kinds of latent distribution to prove if the proposed argument stands. All variants include Known Space Initialization, Unknown Anchor Generation and Centralized Space Refinement stages and only use different loss function (leading to different distribution). A straightforward idea is the opposite of *CSL* that to spread all the unknown further away from the center point than the known samples. We refer to this method as counter-centralized (C-Cent.) space. It can be implemented easily by replacing the $\mathscr {L}_c$ loss with $-\mathscr {L}_c$. We also use the random latent space and uniform space for comparison. In the random space, we only push unknown anchors away from the prototypes, while in the uniform space, we consider the proxy images as a novel class at the Centralized Space Refinement stage to perform prototype learning. The results in Table 4 show that by using a concise and explicit separation, such as C-Cent and *CSL*, the network achieves a solid improvement, proving that our argument stands. As for the deficiency of counter-centralized, we blame it for spreading all unknown samples away from the center point needs to push the unknown samples faster than the known samples. Given the difficulty of manipulating the unknown distribution in latent space, it is also difficult to control the speed of unknown class’ movement in latent space. Thus, the unknown samples may not be effectively separated from the known samples.

**Table 5:** Different $\theta$ value.

$\theta$	AUROC
0.01	0.887
0.1	**0.894**
1	0.892
10	0.885

Significant values are in [bold].

#### Effect of $\theta$

To balance the trade-off between open-set identification and closed-set accuracy, we test the AUROCs with respect to different $\theta$, the results are shown in Table 5. In centralized space refinement, $\theta$ controls the hardness of clustering unknown anchors to the center. On the one hand, when $\theta$ is too small, unknown anchors cannot be effectively clustered to the center point and thus deteriorate the separation ability. On the other hand, given large $\theta$ value, the network tends to focus only on clustering unknown anchors to the center, ignoring the known samples. Thus, the known samples are no longer restricted to the prototypes, closed-set accuracy is damaged.

## Future work

In this paper, the proxy images are generated by a GAN model. According to the most recent researches, diffusion model^34^ has become the state-of-the-art method to generate high quality images^35–37^. The authors believe that it is also possible to train a medical-image-oriented diffusion model, which can synthesize medical images by taking a description of the state of an illness as input. Another possible direction is to train a CLIP model^38^ between medical images and clinical medical records. In this way, we are able to take full advantage of unlabeled medical data. The trained CLIP model can be used as a pre-trained model for the proposed method or achieves open-set recognition in a zero-shot recognition manner.

## Conclusion

In this paper, we present the *Centralized Space Learning* framework for open-set recognition, boosting the robustness of CADs on the recognition/classifition tasks. The proposed centralized space designs an explicit and concise distribution making manipulation on unknown space easier. With the assistance of generated proxy images and unknown anchors, *CSL* surpasses the state-of-the-art methods. With the introduction of *CSL*, we also hope to arouse people’s attention in designing robust and efficient open-set CAD systems. Not only CAD systems, the proposed *CSL* can also be applied to more general open-set recognition tasks. We hope there will also be more general and practical open-set recognition benchmark datasets to evaluate methods in the real target environments.

## Data Availability

The MURA^27^ dataset was released by Stanford University and is available at https://stanfordmlgroup.github.io/competitions/mura/. The ISIC^28^ dataset is available at https://www.isic-archive.com/.
